# Differences in expression of genes related to steroidgenesis in abdominal subcutaneous adipose tissue of pregnant women with and without PCOS; a case control study

**DOI:** 10.1186/s12884-021-03957-5

**Published:** 2021-07-07

**Authors:** Neda Emami, Ashraf Moini, Parichehreh Yaghmaei, Vahid Akbarinejad, Maryam Shahhoseini, AliReza Alizadeh

**Affiliations:** 1 Department of Biology, Faculty of Science, Science and Research Branch, Islamic Azad University, Tehran, Iran; 2 Department of Endocrinology and Female Infertility, Reproductive Biomedicine Research Center, Royan Institute for Reproductive Biomedicine, ACECR, Tehran, Iran; 3 Breast Disease Research Center (BDRC), Tehran University of Medical Sciences, Tehran, Iran; 4 Department of Gynecology and Obstetrics, Arash Women’s Hospital, Tehran University of Medical Sciences, Tehran, Iran; 5 Department of Theriogenology, Faculty of Veterinary Medicine, University of Tehran, Tehran, Iran; 6 Reproductive Epidemiology Research Center, Royan Institute for Reproductive Biomedicine, ACECR, Tehran, Iran; 7 Department of Genetics, Reproductive Biomedicine Research Center, Royan Institute for Reproductive Biomedicine, ACECR, Tehran, Iran; 8 Department of Cell and Molecular Biology, School of Biology, College of Science, University of Tehran, Tehran, Iran; 9 Department of Embryology, Reproductive Biomedicine Research Center, Royan Institute for Reproductive Biomedicine, ACECR, Tehran, Iran

**Keywords:** Polycystic ovary syndrome, Subcutaneous adipose tissue, Sex steroid, Mineralocorticoids, Glucocorticoids

## Abstract

**Background:**

It was reported that steroid-related gene expressions in the adipose tissue (AT) of women differ between women affected with polycystic ovary syndrome (PCOS) and non-PCOS. Although association between PCOS in mother and offspring’s health is a crucial issue, there are few studies focusing on AT of pregnant women suffering from PCOS. Our objectives were to determine the differences between mRNA expression levels of key steroid-converting enzymes in abdominal subcutaneous AT of pregnant women afflicted with PCOS and non-PCOS.

**Methods:**

Twelve pregnant women with PCOS (case) and thirty six non-PCOS pregnant women (control) (1:3 ratio; age- and BMI-matched) undergoing cesarean section were enrolled for the present study. Expressions of fifteen genes related to steriodogenesis in abdominal subcutaneous AT were investigated using quantitative real-time PCR.

**Results:**

No significant differences were detected with respect to age, BMI (prior pregnancy and at delivery day), gestational period and parity among pregnant women with PCOS and non-PCOS. Most of the sex steroid-converting genes except 17β-Hydroxysteroid dehydrogenases2 (*17BHSD2*), were highly expressed on the day of delivery in subcutaneous AT. Women with PCOS showed significantly higher mRNA levels of steroidgenic acute regulator (*STAR; P* < *0.001*), cytochrome P450 monooxygenase (*CYP11A1; P* < *0.05*), 17α-hydroxylase (*CYP17A1; P* < *0.05*), and 11β-Hydroxysteroid dehydrogenase (*11BHSD1* and *11BHSD2; P* < *0.05*). The expression of steroid 21-hydroxylase (*CYP21)* in non-PCOS was fourfold higher than those of women with PCOS (*P* < 0.001). There were no significant differences between relative expression of aromatase cytochrome P450 *(CYP19A1),* 3β-hydroxysteroid dehydrogenase (*3BHSD1 *and *3BHSD2*), and *17BHSD* family (1, 3, 5, 7, and 12) between the two groups.

**Conclusion:**

The expression levels of genes related to sex steroids metabolism were similar to age-matched and BMI- matched pregnant non-PCOS and pregnant women with PCOS at delivery day. However, the alterations in gene expressions involved in glucocorticoids and mineralocorticoids metabolism were shown. It is necessary to point out that further studies regarding functional activity are required. More attention should be given to AT of pregnant women with PCOS that was previously ignored.

**Supplementary Information:**

The online version contains supplementary material available at 10.1186/s12884-021-03957-5.

## Background

Polycystic ovary syndrome (PCOS) is a common endocrine and metabolic disease, occurring in 4–18% of adolescents and women of childbearing age relying upon factors like adiposity and diagnostic criteria [1, 2]. Numerous health issues such as metabolic syndrome and androgen imbalance are the typical problems in women suffering from PCOS [3]. Moreover, recent studies pointed out an increased risk of autism in children of mothers with PCOS [4]. In a depot-specific manner, in conditions of women androgen excess such as PCOS, androgens exert metabolically harmful effects on adipose tissue (AT) function [5]. As a scientific background, it has been suggested that AT contain the steroidogenic machinery necessary for the initiation of steroid biosynthesis de novo from cholesterol [6, 7] which warrants further studies in pregnant women with and without PCOS.

It was proposed that AT have pivotal roles in metabolism of steroid family such as sex steroid, mineralocorticoids, and glucocorticoids [8]. The composition and the concentration of steroids released into circulation from AT are influenced by AT functionality [6, 8] and adipocyte function and possibly affect the size and distribution of AT which can modulated by androgen and cortisol [9, 10]. Sex steroid hormone metabolism has been addressed in research driven by the worldwide human epidemic of adiposities and related disturbances in PCOS with focusing on crucial roles of AT [11, 12]. It was reported that the abnormal conversion of androgen precursors into both active and inactive forms at the receptor level bear promoting roles on the pathogenicity in women with PCOS, being independent of the low amount of androgen or estrogen derived from AT [13, 14]. Meanwhile, Bellemare et al. [15] confimed the critical roles of type 12 17β-hydroxysteroid dehydrogenase (*17BHSD12*) in adipocyte differentiation and Quinkler et al. [16] candidate *17* β-hydroxysteroid dehydrogenase 5 *(17 BHSD5*: account for the conversion of androstenedione into testosterone) in AT as a biomarker for overweight and hyperandrogenism in women with PCOS.

On the other hand, metabolic alteration of glucocorticoids was reported as an effective factor in AT of women with PCOS. In fact, expression of *11BHSD1* mRNA increased in the abdominal subcutaneous AT of women with PCOS, which may contribute to the secretion of local active glucocorticoids (cortisol) [17, 18]. Uniquely, an observation by Mackenzie et al. 2008, was that de novo syntheses of sex steroids, cortisol and aldosterone from cholesterol are not feasible in pregnant women around delivery due to the fact that they cannot reveal effective expression of key steroidogenic mRNAs in the AT on delivery day [19]. Accordingly, Lim et al. [1] showed that women with PCOS have increased risk of metabolic syndrome associated with obesity and metabolic features but not with indices of hyperandrogenism. Since there are many pieces to the puzzle of how the components of PCOS are interrelated, it appears that the crosstalk between several members of storied family have decisive roles in AT, particularly in mother suffering from PCOS. Hence, AT function and metabolic behavior associated with PCOS; however, AT in pregnant women with PCOS have not been considered in previous studies.

AT location (subcutaneous *versus* visceral) has basic roles in AT functionality [8]. Nowadays, Schiffer et al. suggested that adipose androgen generation increase in subcutaneous AT in women with PCOS [5]. The vital roles of subcutaneous AT in women was approved by Kuchenbecker et al. [20] who indicated that the subcutaneous abdominal AT is extremely related with anovulation in women with obesity and infertility. Not only have few research have been conducted about comprehensive assessment of steroid family genes (sex steroid, mineralocorticoids, glucocorticoids) involved in AT’s steroidogenesis in women with PCOS. As far as our knowledge extends, the question of whether these gene expression in AT of pregnant women with PCOS which may differ from that of non-PCOS pregnant women has not been studied yet. One of the prespecified hypothesis was the differences in expression of genes related to cortisol metabolism in AT of mother with PCOS and non-PCOS. With this background, the aims of the current study are investigate the mRNA levels of fifteen steroids genes in subcutaneous AT in pregnant women with and without PCOS.

## Materials and methods

### Subjects and adipose tissue biopsies

Twelve pregnant women with PCOS (case) and thirty six non-PCOS pregnant women (control) (1:3 ratio; age- and BMI-matched) undergoing cesarean section were enrolled for the present study. Abdominal subcutaneous AT samples were collected. Samples and demographic data were collected from three hospitals in Tehran, Iran after obtaining permission from Royan Institute Ethics Committee, Tehran, Iran (IR.ACECR.ROYAN.REC.1398.087). Signed informed consent was obtained from all subjects. In the present research the sample size was estimated 48 women (12 case with 36 controls) with 1:3 ratio without any missing samples. In compliance with the guideline for the assessment and management of PCOS [21], the diagnostic criteria of PCOS are the presence of two or more significant signs of syndrome and patients must met two out of three signs consisting of hyperandrogenism, ovulatory dysfunction, and polycystic ovaries. Diagnosis of PCOS in the enrolled subjects was the responsibility of the medical practitioner affiliated to Royan Institute. Intake of any medication affecting glucose, lipid metabolism and diabetes were defined as exclusion criteria. Gestational diabetes mellitus (GDM), gestational hypertension (HPT) and other metabolic disorders were considered as exclusion criteria. General information such as: age, weight (prior pregnancy and at delivery day), height, gestational information, and habits such as smoking, specific nutritional practices and alcohol consumption before delivery day and on the sampling day was collected applying a written questionnaire. Diagnosis by the medical practitioner and previous caesarean delivery as well as maternal choice (< 10%) were reasons for delivery by caesarean section.

Overall, we tried to ensure that the previous valid indicators are fully adhered to achieve efficient and valid results and the small sample size as a bias were described in study limitations. On the basis of a study launched by Li et al. [17], we considered power or 1-*β* equal to 80% and Type I error rate, *α* equal 5%. It is estimated 12 PCOS with 36 controls (for improving power of the study) with 1:3 ratio without any missing samples. Details of the sample collection were presented by Emami et al. [22]. At the time of caesarean section, the surgeon excised 3–4 g of subcutaneous AT upon exposing the abdominal cavity. Then, the respective biopsies were immediately washed by isotonic saline solution, segmented and floated in liquid nitrogen, placed in cryovial tubes and snap-frozen. Samples were stored at liquid nitrogen (-196 $^\circ$ C) until analysis [19].

### RNA extraction, cDNA synthesis and qPCR procedures

For all samples, total RNA were extracted by the RNeasy lipid tissue mini kit (QIAGEN, Germany). The integrity of the extracted RNA was evaluated using 1% agarose gels and stained with gel red. Spectrophotometer (Bio-Rad, Stanford, USA) used for evaluation of the quality of extracted RNA in terms of the A260/280 ratio. Afterward, PrimeScript RT Reagent Kit (TAKARA, Japan) used for cDNA synthesis on RNA samples, in a way that all experiments included RT controls and negative controls (without cDNA). PCR products were analyzed by gel electrophoresis. By qRT-PCR on the Step-One RT-PCR system (Applied Biosystems, USA), mRNA quantification was conducted and each reaction was run in duplicate. Primer design was performed for all fifteen steroids target genes using the Perl primer Software (version1.1.21) and the NCBI primer Blast. The primer sets, product size, and NIH GenBank accession numbers are listed (Table 1). Melting curve analyses were made to verify primer specificities and standard curves obtained for each gene to evaluate primer efficiency using the logarithmic dilution series of total cDNA. Ultimately, the housekeeping gene *GAPDH* was recruited for normalization and messenger RNA expression levels of all target genes were analyzed by quantitative real‐time PCR (2^−ΔΔ*C*T^).

**Table 1:** Sequences of the primers used for the quantification of the target genes (F: Forward; R: Revers)

Gene	NIH Gen Bank accession No	Product length (bp)	Primer sequence 5´- 3´
*STAR*	NM:000,349.2	114	GCCCAAGAGCATCATCAAC(F) GCTGGTCTTCAACACCTG (R)
*HSD11B1*	NM:001,206,741.1	180	GCATTGTTGTCGTCTCCTCT(F) TGGCTGTTTCTGTGTCTATGAG(R)
*HSD11B2*	NM:000,196.3	162	GCTGTGAACTCCTTCCCT(F) CGATGTAGTCCTTGCCGT(R)
*HSD17B2*	NM:002,153.2	136	TCTGCCTGCTCATCCTGT(F) CAATCACCACCTGTCACCA(R)
*HSD17B3*	NM:000,197.1	138	ATGCTTCCAAACCTTCTCCC(F) GAGACCTTTCTGCCTTGATTCC(R)
*HSD17B5*	NM:001,253,908.1	151	CTCCAGAGGTTCCGAGAA(F) CTCTTCACACTGCCATCTG(R)
*HSD17B7*	NM:001,304,512.1	112	TCATCTGTGTTTGGCGTG(F) GTTGCTGACATCCACCTG(R)
*HSD17B12*	NM:016,142.2	113	CCTACCTAGCCCTGCGTATT(F) ACCTGTGACAACTGCCCA(R)
*HSD3B1*	NM:000,862.2	185	CTTGGTGAAGGAGAAGGAGC(F) AGGCGGTGTGGATGATGA(R)
*HSD3B2*	NM:000,198.3	173	TGCCAGTCTTCATCTACACC(F) TAGATTCCACCCATTAGCCG(R)
*CYP17A1*	NM:000,102.3	154	GATAACCACATTCTCACCACC(F) GGCTGAAACCCACATTCTG(R)
*CYP11A1*	NM:000,781.2	169	CTTCCTTTCTGTCTCAATTCCC(F) TCTACCAGATGTTCCACACC(R)
*CYP21A2*	NM:000,500.8	103	TGAAGCAGGCCATAGAGAAG(F) ATGTAGTCCATCATGTCCCTC(R)
*HSD17B1*	NM:000,413.3	234	TTCAGATCCATCCCAGAGC(F) TTGATGTCCCTTACGTCCAG(R)
*CYP19*	NM: 000,103.4	116	AGGAGGTGACCAATGAATCG (F) CACGATAGCACTTTCGTCCA (R)
*GAPDH*	NM: 001,256,799.3	134	TGAGAAGTATGACAACAGCCTC(F) TGATGGCATGGACTGTGGT (R)

### Statistical analysis

Data were initially tested for normal distribution using Kolmogorov–Smirnov test. Data with normal distribution were analyzed using *t* test. Data that did not have normal distribution were analyzed using Mann–Whitney U test. Our study was conducted without any missing samples. However, *17BHSD2* was undetectable in AT of both groups with the protocol used herein and we could not report any data regarding this target gene. All analyses were made in SAS. The differences were taken into account significant at *P* ≤ 0.05.

## Results

### Characteristics of PCOS patients and non-PCOS pregnant women

No significant differences were detected with respect to age, BMI (prior pregnancy and at delivery day), gestational period and parity among 36 non-PCOS and 12 PCOS pregnant women (Table 2). We access to all mentioned demographic data of all participants.

**Table 2:** General characteristics of study participants (PCOS: polycystic ovary syndrome and non-PCOS) (mean ± sd) (ns.; Non-significant; *p* > 0.05)

	non-PCOS (*n* = 36)	PCOS (*n* = 12)	*P*-Value
**Age** (year)	32 ± 5.7	31.6 ± 3.6	ns
**BMI** (kgm^−2^) prior pregnancy	25.4 ± 6.06	26.2 ± 6.11	ns
**BMI** (kgm^−2^) at delivery day	31 ± 5.13	30.5 ± 5.74	ns
**Gestation period** (Day)	264 ± 13	267 ± 5	ns
**Parity** (no.)	1.4 ± 0.62	1.4 ± 0.51	ns

### Profiling of the key steroidogenic genes in subcutaneous AT

**Fig. 1:**
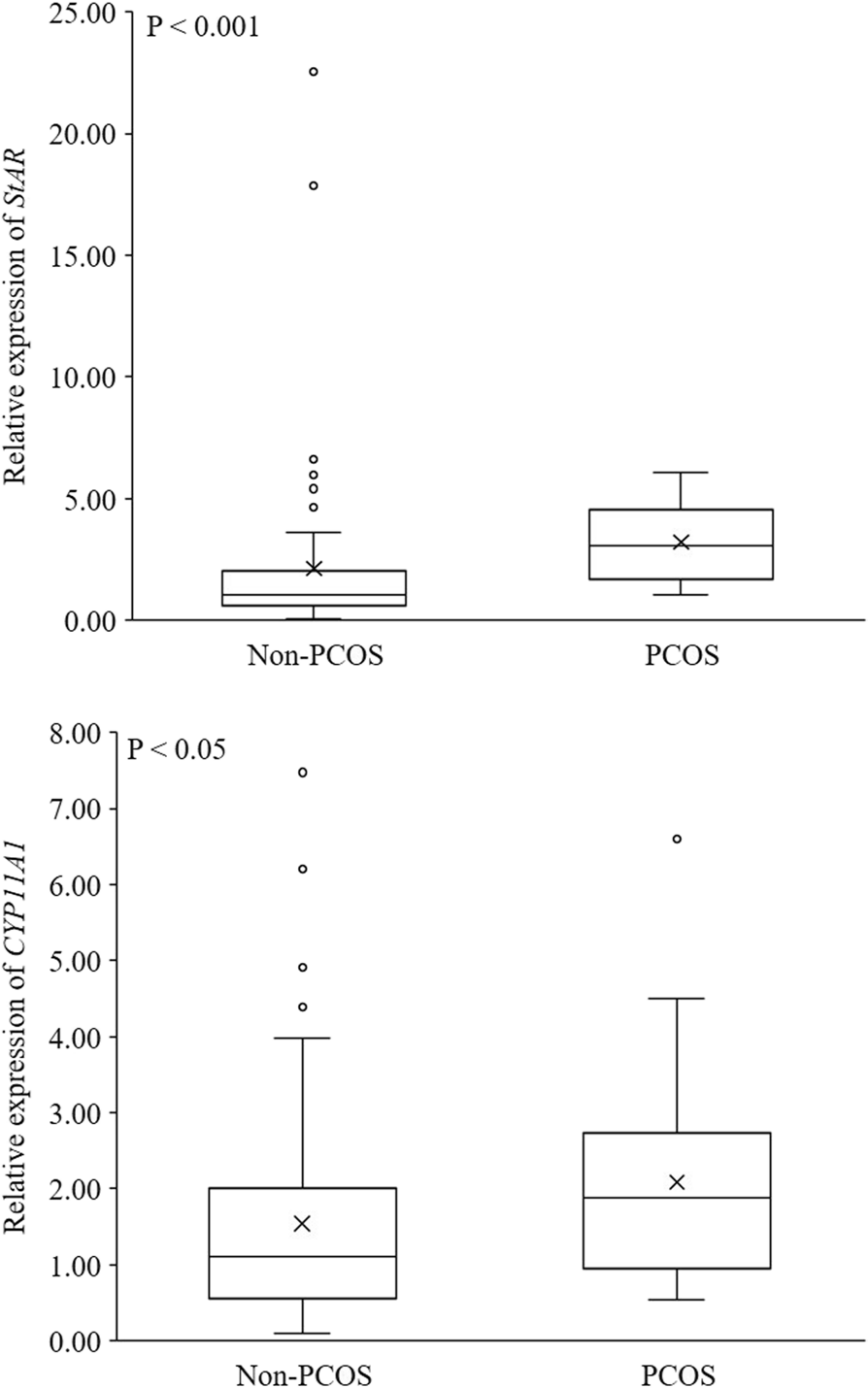
mRNA relative expression of the key enzymes involved in the initial steps of steroidogenesis in adipose tissue based on qRT-PCR in non-PCOS pregnant women (*n* = 36) and PCOS (*n* = 12) pregnant women

**Fig. 2:**
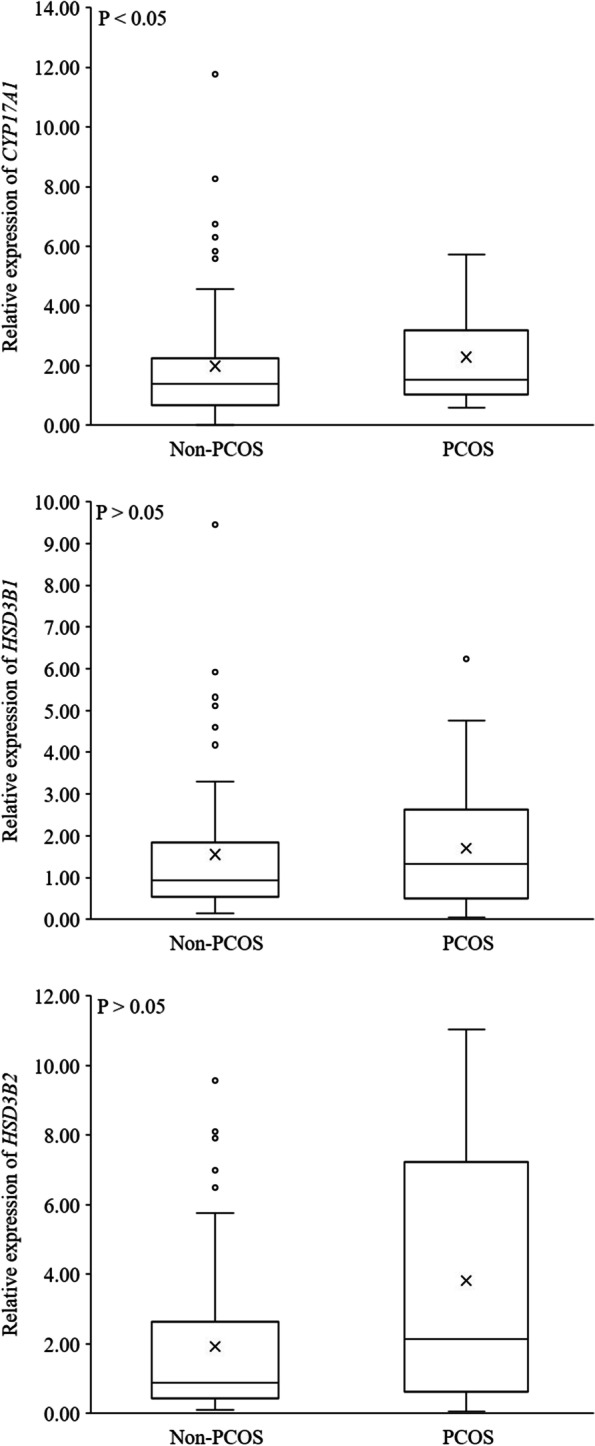
mRNA relative expression of *CYP17*, *3BHSD1* and *3BHSD2* in adipose tissue based on qRT-PCR in non-PCOS pregnant women (*n* = 36) and PCOS (*n* = 12) pregnant women

**Fig. 3:**
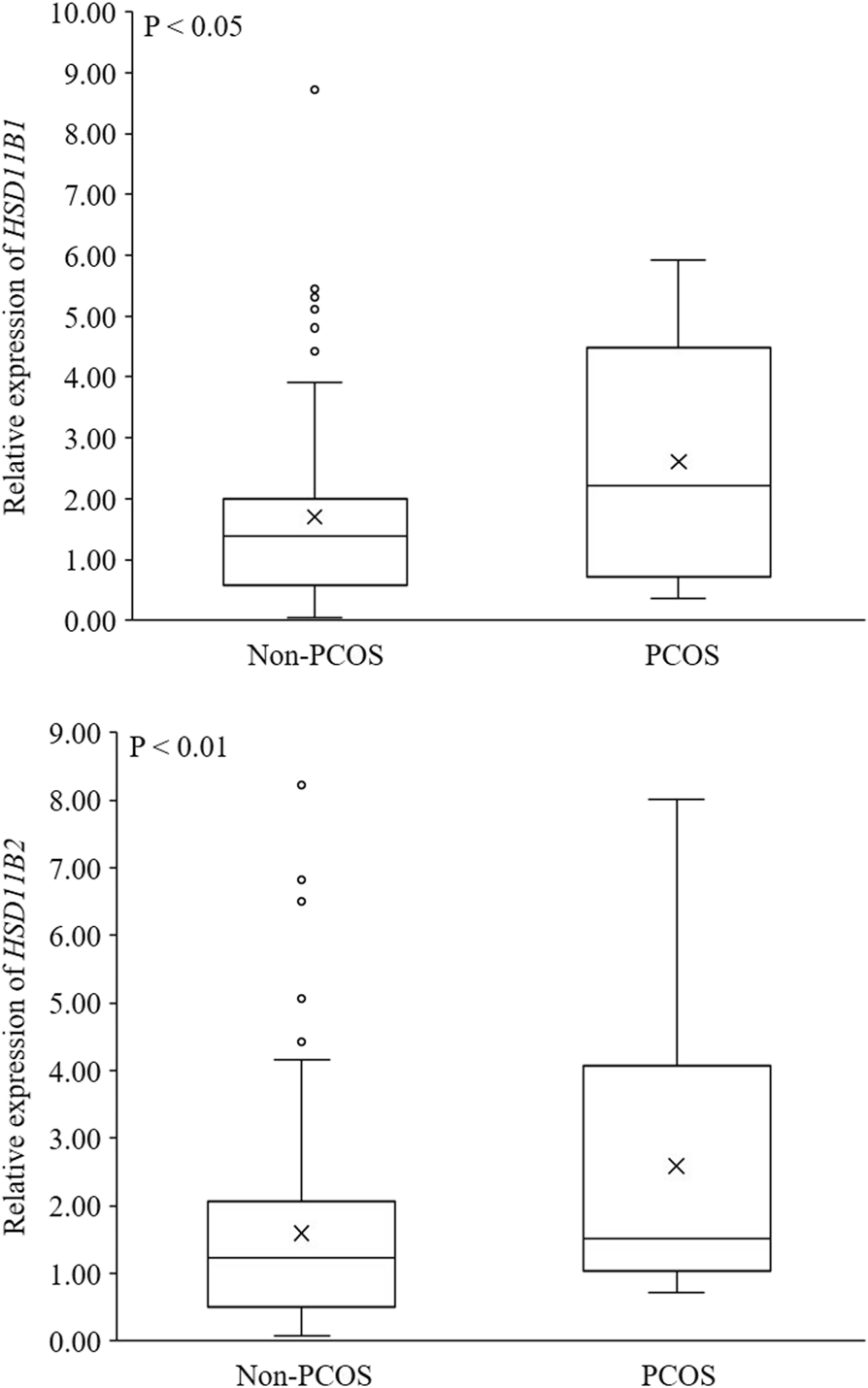
mRNA relative expression of *11BHSD1* and *11BHSD2* which related to glucocorticoids metabolism in adipose tissue based on qRT-PCR in non-PCOS pregnant women (*n* = 36) and PCOS (*n* = 12) pregnant women

**Fig. 4:**
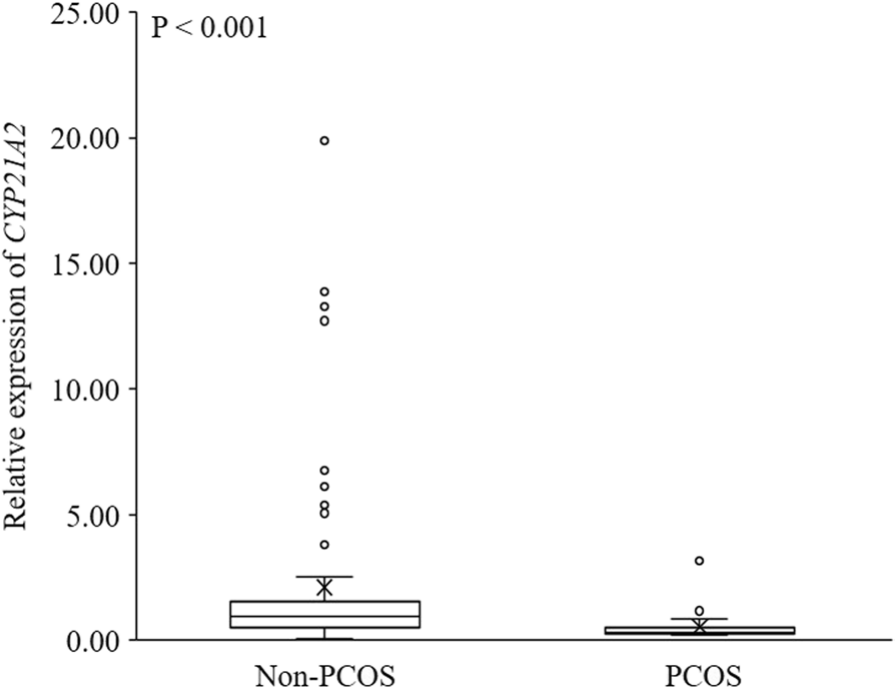
mRNA relative expression of *CYP21* which related to mineralocorticoids metabolism in adipose tissue based on qRT-PCR in non-PCOS pregnant women (*n* = 36) and PCOS (*n* = 12) pregnant women

**Fig. 5:**
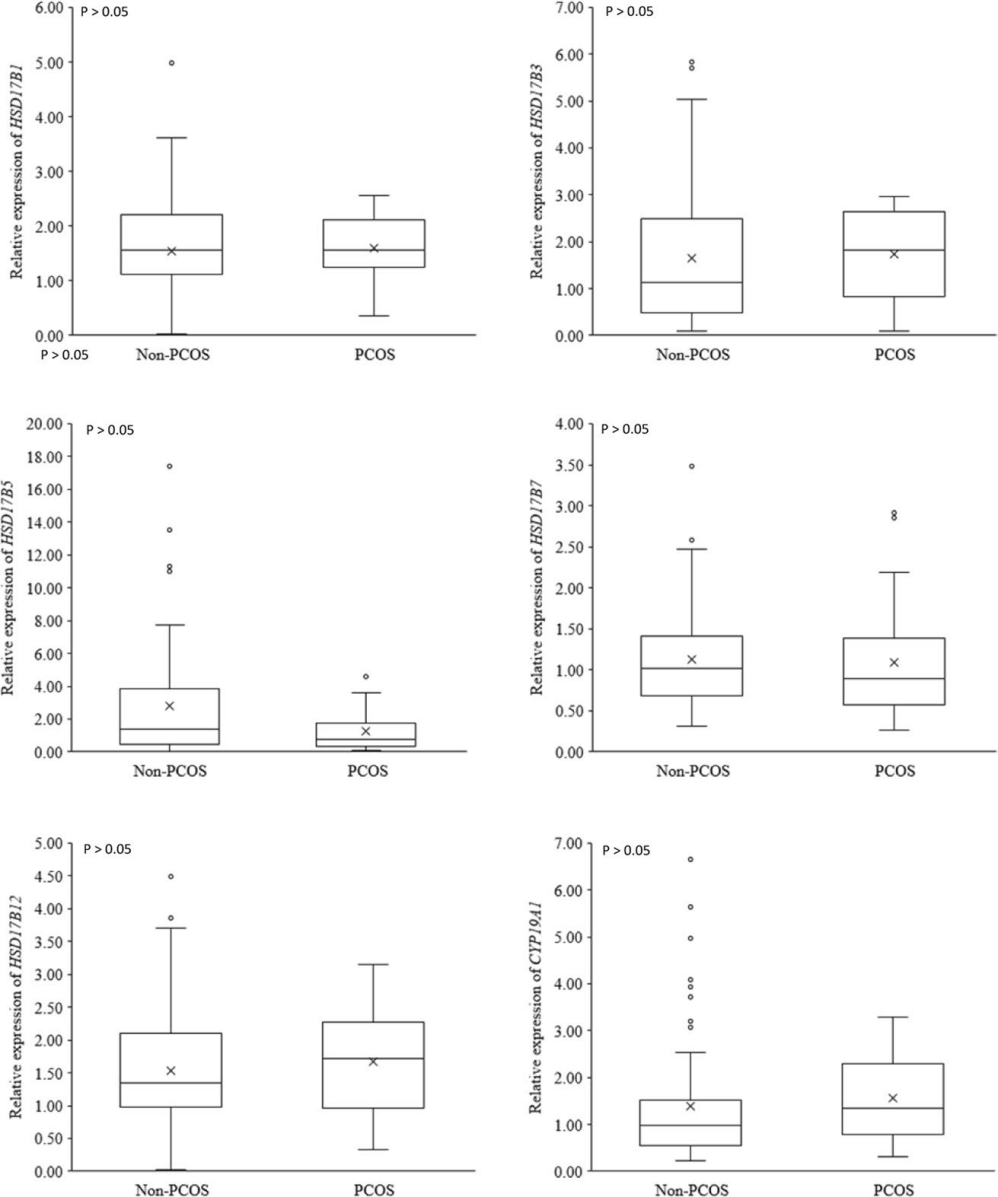
mRNA relative expression of genes involved in sex steroid metabolism (*17BHSD* family and *CYP19*) in adipose tissue based on qRT-PCR in non-PCOS pregnant women (*n* = 36) and PCOS (*n* = 12) pregnant women

The expression profile of key steroidogenic enzyme was compared in human AT obtained from pregnant women with PCOS and non-PCOS (Table 3). mRNAs of *STAR* (*P* < 0.001) and *CYP11A1* (*P* < 0.05) – the genes involved in the initial phase of steroidogenesis – were substantially significantly more ample in the subcutaneous AT of women with PCOS, than in those of non-PCOS (Fig. 1). Furthermore, *CYP17A1* mRNA changed in the pregnant women with PCOS, being significantly (*P* < 0.05) higher than that of non-PCOS group (Fig. 2). The mRNA encoding *11BHSD1* (*P* < 0.05) and *11BHSD2* (*P* < 0.01), the genes related to cortisol metabolism, were more abundant in women with PCOS than non-PCOS (Fig. 3). Conversely, the expression of *CYP21A2*, the gene involved in the mineralocorticoids metabolism, was fourfold higher in non-PCOS group than that of the PCOS group (Fig. 4; *P* < 0.001). There were no significant differences between relative expression of *3BHSD1* and *3BHSD2* (Fig. 2). There were also no differences between the groups in the expressions of genes related to sex steroid metabolism such as the *CYP19A1,* and *17BHSD* families (1, 3, 5, 7, and 12) (Fig. 5). Expression of *17BHSD2* was undetectable in AT both groups with focusing on the protocol used herein.

**Table 3:** Levels of mRNA in subcutaneous adipose tissue samples of PCOS (*n* = 12) relative to non-PCOS (*n* = 36) (ns.: non-significant)

Gene	Relative level	Fold difference	***P*-Value**
*STAR*	PCOS > non-PCOS	1.5	< 0.001
*CYP11A1*	PCOS > non-PCOS	1.3	< 0.05
*HSD11B1*	PCOS > non-PCOS	1.5	< 0.05
*HSD11B2*	PCOS > non-PCOS	1.6	< 0.01
*CYP17A1*	PCOS > non-PCOS	1.2	< 0.05
*CYP21A2*	non-PCOS > PCOS	4	< 0.001
*HSD17B1*	PCOS > non-PCOS	1.03	ns
*HSD17B3*	PCOS > non-PCOS	1.05	ns
*HSD17B5*	non-PCOS > PCOS	2.2	ns
*HSD17B7*	non-PCOS > PCOS	1.02	ns
*HSD17B12*	PCOS > non-PCOS	1.09	ns
*HSD3B1*	PCOS > non-PCOS	1.09	ns
*HSD3B2*	PCOS > non-PCOS	2	ns
*CYP 19*	PCOS > non-PCOS	1.12	ns
*HSD17B2*	undetectable	-	-

## Discussion

The present study provides noticeable differences between expression of genes involved in initial steps of steroidogenesis, glucocorticoids and mineralocorticoids metabolism in AT of pregnant women with PCOS and non-PCOS, whereas expression of genes related to sex steroid metabolism had similar in case and control groups. Our experiment has a few constraints. Small sample size was the restriction of the current study for age- and BMI- matched women with PCOS. However, we worked on samples collected from 48 Iranian mothers (PCOS and non-PCOS), while former studies [12, 17, 18, 23, 24] conducted on gene expression in AT samples of non-pregnant women with PCOS have similar or lower sample size or assessed few genes. It was attempted select subjects with similar BMI and age, with no metabolic disorders and with approximately healthy lifestyle. It would have been interesting to compare expressions across gestation as well as further studies with larger sample sizes which could confirm these results in pregnant women with PCOS alongside measured several hormone levels. Our first objective was to check the expression pattern of major genes involved in steroidogenesis. However, it is better that some clustering of genes functioning in various steroid hormone subtypes, particularly regarding pregnancy state exclusively will be investigated in future studies. Other factors such as AT location may be associated with the detected data which requires further study for several locations (subcutaneous *versus* visceral) in mother with PCOS.

It was reported that both *STAR* and *CYP11A1* are rate-restricting factors for steroidogenesis as they produce crucial precursors and this pathway initiated by *STAR* and *CYP11A1* [6, 25]. Not only the total amount of steroids within AT was found to be 40–400 times greater than in plasma, but there was also a positive gradient between tissue and plasma [12]. Former research have indicated the relation between *CYP11A1* and PCOS [26]. For the first time in 2008, MacKenzie et al. [19] detected *STAR* and *CYP11A1* gene expression in paired visceral and subcutaneous AT from the lower abdominal region taken from eight women undergoing caesarean section. Accordingly, Wang et al. [12] revealed expression of *CYP11A1* in abdominal subcutaneous AT taken from non-pregnant women. Although Alizadeh et al. [27] reported that *CYP11A1* mRNA was not detectable in retroperitoneal and subcutaneous dairy cattle AT, they showed that the amount of *STAR* mRNA in subcutaneous AT on the day of delivery was threefold higher than that of subjects before calving. In the present study, the up-regulation of genes involved in initial steps of steriodogenes on the day of delivery in mothers with PCOS show a rise in capacity for cholesterol uptake to the inner mitochondrial membrane and steroidogenesis stimulation. Hence, women with PCOS may have more functional and effective sources of steroid metabolism pathways in subcutaneous AT on the day of delivery than age- and BMI-matched non-PCOS.

*CYP17A1* is a key step point in steroid biosynthesis, driving the pathway to the direction of either sex steroid, mineralocorticoid and glucocorticoid production or metabolism [28]. It was reported that *CYP17A1* mRNA was more ample in ovarian theca cells of women with PCOS than those of non-PCOS [28, 29]. Likewise, *CYP17A1* was transcribed higher in subcutaneous AT of women with PCOS than non-PCOS. MacKenzie et al. did not find out this gene in subcutaneous AT on the day of delivery, and neither did Dalla Valle et al. in AT of non-pregnant women [19, 30]. However, the presence of *CYP17A1* gene in subcutaneous AT of women with PCOS was reported by Wang et al. [12]. Uniquely, Kinoshita et al. [31] detected CYP17A1 activity at the protein level in women AT. In fact in the case of affected with PCOS, a potential for up-regulation of *CYP17A1* expression in AT was proposed that may contribute to hyperandrogenism [32–34]. Thus, our data in AT for CYP17A1 support the theory [28, 29, 34] that overexpression of this gene playing a critical role in pregnant women with PCOS, which may associated with the regulation of glucocorticoids and mineralocorticoids metabolism.

Among fourteen genes detected and compared among both groups, only relative expression of *CYP21* was fourfold higher in non-PCOS than in PCOS. Similarly, Azziz et al. [35] and Witchel and Aston [36] revealed the deficiency of 21-hydroxylase in hirsute women with PCOS. The 21-hydroxylase enzyme encoded by *CYP21* has pivotal roles and one of the main steps in adrenal and ovarian steroidogenesis is the conversion of 17-hydroxyprogesterone into 11-deoxycortisol, catalyzed by *CYP21*. For most cases of congenital adrenal hyperplasia, the deficiency of this enzyme, inherited by an autosomal recessive trait, is a typical sign, subsequently increased serum 17-hydroxyprogesterone levels correlated with its deficiency. Moreover, patients afflicted with heterozygote *CYP21* mutations as well as clinical symptoms exhibit PCOS-like phenotype [37]. Thus, the alteration in amounts of *CYP21* mRNA in AT support the above mentioned reasoning.

Our data confirmed the achievements of former investigations [10, 17, 18, 23, 24] by several BMI ranges that not only *HSD11B1* and *HSD11B2* mRNA are present in both subjects but also the expression of *HSD11B1* are more in subcutaneous AT of women with PCOS than non-PCOS. It is obviously pointing out that a rise in local production of cortisol might have a role in the several disorders found in women with PCOS [18]. On the other hand, due to highest level of cortisol around delivery day and pivotal roles of cortisol during pregnancy, more attention must be paid to cortisol metabolism in mother with PCOS. Mackenzie et al. [19] in human and Alizadeh et al. [38] in dairy cattle pointed out the high expression of *HSD11B1* gene in both subcutaneous and visceral AT on the day of delivery. On the basis of the evidence for *HSD11B1* in human AT and bovine AT from different visceral and subcutaneous depots, AT probably influence glucocorticoid metabolism with consequences for both endocrine as well as auto/paracrine glucocorticoid effects locally within AT, the latter being supported by the presence of glucocorticoid receptors, with demonstration in bovine AT [39]. The increasing wave of cortisol metabolism around parturition is normal; however our study obviously revealed 1.5-fold elevation of *HSD11B1* mRNA levels in women with PCOS over non-PCOS at delivery day. Thus, we encounter with the rise of *HSD11B1* in women with PCOS alongside with the unavoidable rise of *HSD11B1* around delivery day. In general, our achievements in pregnant women with PCOS cast more lights on the emerging to note the cortisol metabolism in mother suffering from PCOS.

The presence of *CYP19* (aromatase) mRNA in human AT has already been well established by Corbould et al. [40] and Mackenzie et al. [19]. Although it has been proposed that PCOS may result from reduced aromatase activity and subcutaneous AT have higher expression of *CYP19* in comparison to visceral AT in morbidly obese men and premenopausal women [41], we cannot detect difference in mRNA levels of *CYP19* between women with PCOS and non-PCOS. Formerly, the conversion of androstenedione to estrone in subcutaneous AT from the lower body of women (i.e., thighs and buttocks) was shown to be higher than that of in the subcutaneous AT of upper body fat (i.e., breast and abdomen), along with the highest level of *CYP19* gene expression [42]. Altogether, the lack of changes in *CYP19* suggest that delivery day or sampling area may influence *CYP19* expression than PCOS status.

Enzymes with activities related to different *17BHSD* isoforms are widespread in classic steroidogenic tissues such as the testis, ovary, and placenta as well as in a several peripheral sites including AT [43]. The conversion of estrone to estradiol catalyzed by the estrogenic isoforms of *17BHSD* (types 1, 7 and 12) [6] and they are similar in PCOS and non-PCOS groups in the present study. It seems that subcutaneous AT may serve as a dominant activation site for androgens than estrogens, because the *17BHSD* family genes involved in the estrogenic process in AT are much fewer than those of the androgenic one. Bellemare et al. [15] suggested that *17BHSD12* be involved in the local conversion of estrone into estradiol in differentiated adipocytes and it has more activity than types 1 and 7. Uniquely, Quinkler et al. [16] pointed out that *17BHSD5* has crucial roles in women androgen metabolism in subcutaneous buttock AT. Therefore, it is expected that some members of *17BHSD* family, particularly *17BHSD5* and *17BHSD12* may differ between experimental groups; however, our achievements did not support the hypotheses concerning different sex steroid related gene expression in subcutaneous AT of women with PCOS on delivery day. While it appears that sampling area (buttock AT *vs.* abdominal AT) may influence these results, but the mRNA levels of *17BHSD5* were 2-folds higher in abdominal subcutaneous AT of women with PCOS in comparison to the non-PCOS [12]. Therefore, this result may affect the status on the day of delivery, as indicated by previous study. Although we identified the expression of genes related to sex steroid metabolism in AT of pregnant women with and without PCOS, Mackenzie et al. [19] suggested that de novo synthesis of sex steroids from cholesterol be not possible in pregnant women on the day of delivery owing to the lack of key steroidogenic mRNAs in the AT.

Ultimately, in order to generalize our research, the survey on mother with PCOS and offspring health is considered as a spectacular and emerging issue for clinic and basic. Uniquely, Katsigianni et al. [44] and Cherskov et al. [45] reported that children of mothers with PCOS have an higher risk of developing autism spectrum disorder. They supported the theory regarding early life androgen exposure may be important for the development of autism in both sexes supported by Robinson et al. [4]. Our findings about alteration genes involved in cortisol and aldosterone metabolism, suggest further studies are warranted in order to confirm AT’s roles in mother with PCOS concerning metabolism of all steroid family members.

## Conclusion

In conclusion, our data revealed that in AT of pregnant women suffering from PCOS, the genes related to initial steps of steroidogenesis (*STAR* and *CYP11A1*) were more expressed in comparison to age- and BMI-matched non-PCOS. The expression levels of genes related to cortisol metabolism (*11BHSD1* and *11BHSD2*) were higher in women with PCOS than age- and BMI- matched non-PCOS, the profile of which may result from the regulation of glucocorticoid metabolism. This achievement opens horizon for more exploration regarding AT roles in cortisol metabolism in mothers with PCOS. Furthermore, extreme reduction of *CYP21* mRNA in AT confirms gene expression disorders in mineralocorticoid metabolism for women with PCOS. Generally, the alterations in gene expressions involved in glucocorticoids and mineralocorticoids metabolism was indicated which requires further studies with regards to functional activity.

## Supplementary Information

**Additional file 1**. Gene expression data

## Data Availability

All data and material used and/or analyzed during the current study are available from the corresponding authors upon reasonable request. The online version contains supplementary material available.

## Abbreviations

AT: Adipose tissue
PCOS: Polycystic ovary syndrome
*17BHSD*: 17β-Hydroxysteroid dehydrogenases
*STAR*: Steroidgenic acute regulator
*CYP11A1*: Cytochrome P450 monooxygenase
*CYP17A1*: 17α-Hydroxylase
*11BHSD*: 11β-Hydroxysteroid dehydrogenase
*CYP21*: Steroid 21-hydroxylase
*CYP19A1*: Cytochrome P450 aromatase
*3BHSD*: 3β-Hydroxysteroid dehydrogenase
